# Genetically Programmed Single‐Component Protein Hydrogel for Spinal Cord Injury Repair

**DOI:** 10.1002/advs.202405054

**Published:** 2025-01-10

**Authors:** Yi Wei, Xiaolin Zhou, Zhenhua Li, Qing Liu, Han Ding, Yunlong Zhou, Ruo‐feng Yin, Lifei Zheng

**Affiliations:** ^1^ Wenzhou Institute University of Chinese Academy of Sciences Wenzhou Zhejiang 325001 China; ^2^ China‐Japan Union Hospital Jilin University Changchun Jilin 130031 China

**Keywords:** CsgA, hydrogel, recombinant protein, self‐assembly, spinal cord injury repair

## Abstract

Protein self‐assembly allows for the formation of diverse supramolecular materials from relatively simple building blocks. In this study, a single‐component self‐assembling hydrogel is developed using the recombinant protein CsgA, and its successful application for spinal cord injury repair is demonstrated. Gelation is achieved by the physical entanglement of CsgA nanofibrils, resulting in a self‐supporting hydrogel at low concentrations (≥5 mg mL^−1^). By leveraging the programmability of the CsgA gene sequence, the bioactive hydrogel is enhanced by fusing functional peptide GHK. GHK is recognized for its anti‐inflammatory, antioxidant, and neurotrophic factor‐stimulating properties, making it a valuable addition to the hydrogel for spinal cord injury repair applications. In vitro experiments demonstrate that the CsgA‐GHK hydrogel can modulate microglial M2 polarization, promote neuronal differentiation of neural stem cells, and inhibit astrocyte differentiation. Additionally, the hydrogel shows efficacy in alleviating inflammation and promotes neuronal regeneration at the injury site, leading to significant functional recovery in a rat model with compression injury spinal cord cavity. These findings lay the groundwork for developing a modular design platform for recombinant CsgA protein hydrogels in tissue repair applications.

## Graphical abstract

A single‐component self‐assembling hydrogel using recombinant protein CsgA is developed for spinal cord injury repair. Gelation occurs through CsgA nanofibril entanglement, and the hydrogel is enhanced by fusing the GHK peptide. The CsgA‐GHK hydrogel modulates microglial M2 polarization, promotes neural stem cell differentiation, and reduces inflammation, leading to significant functional recovery in a rat model of spinal cord injury.

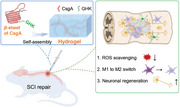

## 1 Introduction

Spinal cord injury (SCI), as a disease of the central nervous system (CNS), often leads to permanent paraplegia, and causes great physical pain and economic costs for patients.^[^ ^1^ ^]^ Pathophysiologically, the occurrence of SCI includes primary injury and secondary injury. The former is usually an external mechanical trauma which permeabilizes neurons and glia to trigger inflammatory and cytotoxic factors. Subsequently, the progressive cell death and spinal cord damage occurs as a secondary injury cascade.^[^ ^2^ ^]^ As an innate defense response, inflammatory response is considered to be an important factor in secondary injury after SCI.^[^ ^3^, ^4^ ^]^ Moreover, CNS has limited axon regeneration and neuronal replenishment capabilities, making the detrimental effects of inflammation more pronounced in this context.^[^ ^5^ ^]^ As a result, glial scars and cystic cavities are formed, which substantially hinder axon regeneration and disrupt nerve signal transmission across the lesion.^[^ ^6^, ^7^ ^]^ Traditional treatments including surgery and drugs (e.g., glucocorticoids and gangliosides) have demonstrated limited efficacy in SCI clinical trials.^[^ ^8^, ^9^ ^]^ Hence, there is an urgent need for innovative and effective therapeutic approaches through modulating the oxidative microenvironment, enhancing neuronal regeneration, and promoting functional recovery.

Hydrogels have been widely explored for nerve injury repair, spanning from natural tissue components to synthetic bio‐based materials.^[^ ^10^ ^]^ Biologically derived materials, such as acellular tissues^[^ ^11^ ^]^ and extracellular matrix‐derived macromolecules like collagen,^[^ ^12^ ^]^ chitosan,^[^ ^13^ ^]^ silk fibroin^[^ ^14^ ^]^ and hyaluronic acid,^[^ ^15^ ^]^ have been extensively utilized. These materials are non‐toxic, readily available, and possess inherent biological activity. However, materials of biological origin carry the risk of batch‐to‐batch variability.^[^ ^16^ ^]^ In contrast, recombinant proteins, synthetic peptides and nucleic acid offer precise sequences and standardized methods of production while retaining the excellent merits of natural materials.^[^ ^17^, ^18^, ^19^ ^]^ They can be easily obtained through genetic and chemical engineering processes. Particularly, recombinant protein hydrogels are superior in terms of economy and diversity.^[^ ^20^, ^21^, ^22^, ^23^ ^]^ Currently, hydrogel networks based on recombinant proteins crosslinked by multi‐arm polyethylene glycol (PEG) and poly (*N*‐isopropyl acrylamide) (PNIPAM) have shown promising therapeutic effects in spinal cord injury repair.^[^ ^24^ ^]^ Nevertheless, these materials necessitate an additional step of chemical cross‐linking and thus may pose biosafety concerns related to the usage of chemical components. A pure protein hydrogel system with bio‐programmability for SCI repair remains elusive.

CsgA, the primary structural component of curli nanofibers found in *Escherichia coli* biofilms, is renowned for its β‐sheet structure, enabling self‐assembly into nanofibers.^[^ ^25^, ^26^, ^27^ ^]^ These structural amyloid CsgA nanofibers are genetically engineerable proteins, allowing for designed functionalization with various heterologous peptides or protein domain inserts without sacrificing their self‐assembling capability into nanofiber structures.^[^ ^28^, ^29^, ^30^ ^]^ For example, fusion proteins combining mussel foot proteins and CsgA have been exploited to create high‐performance underwater adhesives.^[^ ^31^ ^]^ Moreover, an “engineered living materials” concept utilizing *E. coli* with CsgA as the primary structural component has been demonstrated.^[^ ^32^, ^33^, ^34^ ^]^ Trefoil factors known for enhancing intestinal barrier function and epithelial restitution were fused to CsgA to fabricate a live bacteria hydrogel crosslinked by surfactant, showing promising results in treating inflammatory bowel disease.^[^ ^35^, ^36^ ^]^

**Figure 1:**
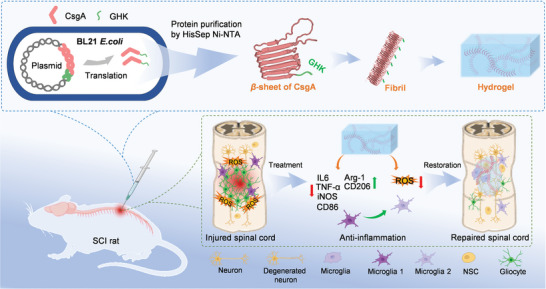
Schematic representation of the CsgA‐GHK self‐assembling fibril‐based hydrogel for SCI repair. The production of CsgA‐GHK recombinant protein showcases genetic programmability, with gelation achieved through the physical entanglement of protein fibers at precise concentrations. Upon implantation at SCI sites, the CsgA‐GHK hydrogel facilitates microglial M2 polarization, scavenges reactive oxygen species (ROS), and mitigates inflammation, leading to neural regeneration, reduction of glial scar formation, and improvement in locomotor function in rat models.

Given its genetic programmability and self‐assembling ability into nanofibers, a single‐component recombinant protein hydrogel based on CsgA is fabricated in this study using a facile preparation method. Gelation is achieved through the physical entanglement of protein fibers at specific concentrations. To equip the hydrogel with anti‐inflammatory properties, human copper‐binding peptide GHK (glycyl‐L‐histidyl‐L‐lysine) is fused to CsgA, since GHK has been recognized for its protective and regenerative capabilities by reducing inflammation and free radical damage (**Figure** **1**).^[^ ^37^, ^38^ ^]^ In vitro experiments conducted with a mouse microglia BV2 cell line show that CsgA‐GHK gel can modulate the oxidative microenvironment of cells and significantly promote anti‐inflammatory M2 microglial polarization. Furthermore, it exhibits the capacity to promote neuronal differentiation of neural stem cells (NSCs). Subsequent in vivo studies demonstrate that the implantation of CsgA‐GHK hydrogel significantly improves functional recovery of rats following SCI. We, therefore, anticipate that this work may pave the way for designing functional gene‐programmed protein self‐assembly hydrogels in the realm of tissue repair and regeneration (Figure 1).

## 2 Results and Discussion

### 2.1 Preparation and Characterization of CsgA and CsgA‐GHK Hydrogels

**Figure 2:**
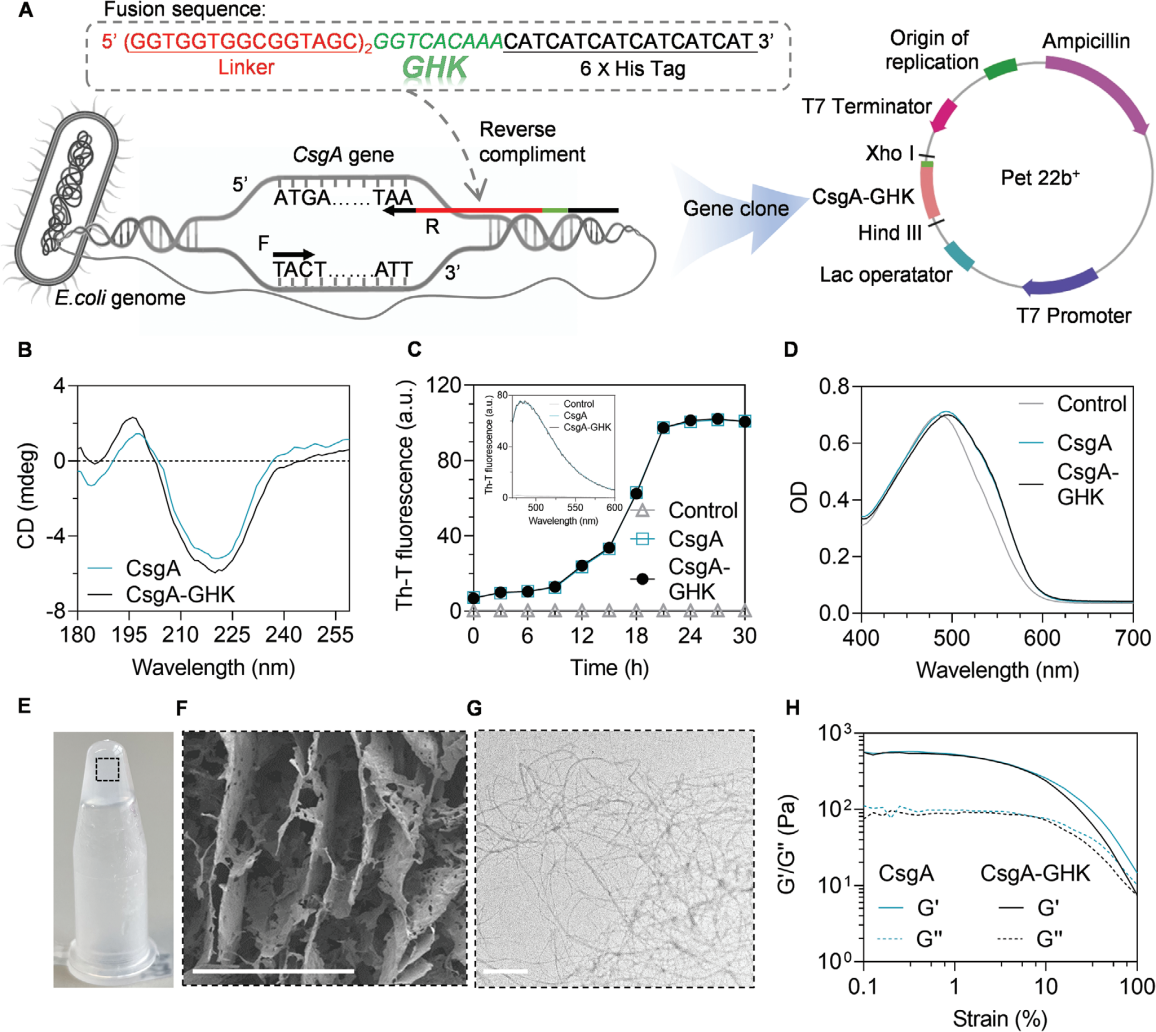
Characterization of CsgA and CsgA‐GHK hydrogels. A) Illustration of the construction of the expression vectors. B) CD spectra of recombinant CsgA and CsgA‐GHK proteins. C) Evaluation of the time‐dependent fluorescence evolution of ThT in the presence of CsgA or CsgA‐GHK, demonstrating the successful formation of fibrils. Insert indicates the fluorescence spectra. A plain buffer was used as a control. (protein concentration: 5 mg mL^−1^; ThT concentration: 1 × 10^−2^ mm). D) UV–vis spectra of CR in the presence of CsgA or CsgA‐GHK. E) Representative photograph of bulk CsgA‐GHK in the gel state. F) SEM image of CsgA‐GHK hydrogel. Scale bars: 100 µm. G) TEM image of CsgA‐GHK nanofiber. Scale bars: 100 µm. H) Rheological characterization of the protein hydrogels.

To obtain the recombinant CsgA and CsgA‐GHK proteins, pET22b^+^‐CsgA and pET22b^+^‐CsgA‐GHK expression vectors were constructed for protein expression in *E. coli* BL21 strain (**Figures** **2**A and S1A,B, Supporting Information). The proteins were purified using a His‐tag purification method. The purities of the recombinant CsgA and CsgA‐GHK proteins were assessed using sodium dodecyl sulfate sulfate‐polyacrylamide gel electrophoresis (SDS‐PAGE) coomassie staining. Both purified proteins displayed a single band with a size of approximately 17 kDa (Figure S1C,E, Supporting Information). To confirm the accuracy of the target bands, anti‐His antibody was used in Western blotting (WB) analysis, which further confirmed the presence of the purified CsgA and CsgA‐GHK proteins (Figure S1D,F, Supporting Information). Additionally, circular dichroism (CD) spectroscopy was utilized to analyze the structures of the CsgA and CsgA‐GHK proteins. As shown in Figure 2B, both proteins exhibited similar positive peaks at 195 nm and negative peaks at 220 nm, indicating that the fusion of GHK displayed negligible influence on the formation of a β‐sheet structure.

Next, nanofiber formation of the CsgA or CsgA‐GHK in solution was investigated using a Thioflavin T (ThT) assay. As shown in Figure 2C, Upon the addition of ThT dye to CsgA or CsgA‐GHK solutions, the fluorescence intensities increased over time, displaying sigmoidal curves with distinct lag, growth, and stationary phases (Figure 2C). The lag phases of both proteins were nearly identical, with all transitioning to the growth phase at 9 h. Moreover, Congo red (CR) binding was observed for CsgA or CsgA‐GHK protein solutions, leading to a distinct red shift in the CR absorption spectrum (Figure 2D). This shift is also indicative of the formation of fibrous structures. The nanofiber structures of CsgA and CsgA‐GHK were directly visualized by transmission electron microscopy (TEM, (Figure 2G and Figure S3C, Supporting Information)).

Subsequently, hydrogels were prepared from the stock solutions of 5 mg mL^−1^ CsgA or CsgA‐GHK proteins (Figure S2, Supporting Information). The gelation was allowed to proceed for 48 h, and subsequent centrifugation was employed to remove excess water, resulting in the final hydrogels (Figure 2E and Figure S3A, Supporting Information). The porous structure of the obtained hydrogels was revealed through scanning electron microscopy (SEM) analysis (Figure 2F and Figure S3B, Supporting Information). Furthermore, the mechanical properties of CsgA and CsgA‐GHK hydrogels were thoroughly investigated by rheological tests. The results of the rheological strain sweep measurements indicated that the storage modulus (*G*') remained around 500 Pa, while the loss modulus (*G*″) was approximately 100 Pa. These values showed high consistency as the applied strain increased from 0.1% to 1%. Then, both of *G*’ value and *G*’’ value were dropped when the strain was increased to 10%, with a crossover point occurring at around 100% strain (Figure 2H). It was reported that the storage modulus and loss modulus of the spinal cord, obtained using dynamic time sweeping mode, were approximately 200 and 50 Pa, respectively, which closely aligns with the current hydrogels.^[^ ^13^ ^]^ We also measured the frequency‐dependent rheological properties of CsgA and CsgA‐GHK hydrogels at a fixed strain of 10% over time. It was found that the storage modulus (*G*’) was always greater than the loss modulus (*G*″) and both were almost independent of frequency (Figure S4A,B, Supporting Information). Additionally, we conducted a continuous step change of shear strain between 1% and 100% for three cycles, with a 2 min recovery time in each cycle, to evaluate the strain‐induced damage and healing of the hydrogels (Figure S4C,D, Supporting Information). The CsgA and CsgA‐GHK hydrogels demonstrated a repeatable restoration of their rheological properties following the continuous step strain, indicating their ability to spontaneously restore structure after damage, although the modulus of the hydrogel decreased after three cycles. Since our hydrogel network relies solely on noncovalent forces, a certain recovery time is necessary after the hydrogel network fails under high shear force.

### 2.2 Evaluation of the Anti‐Inflammatory Effect of CsgA‐GHK Hydrogel

**Figure 3:**
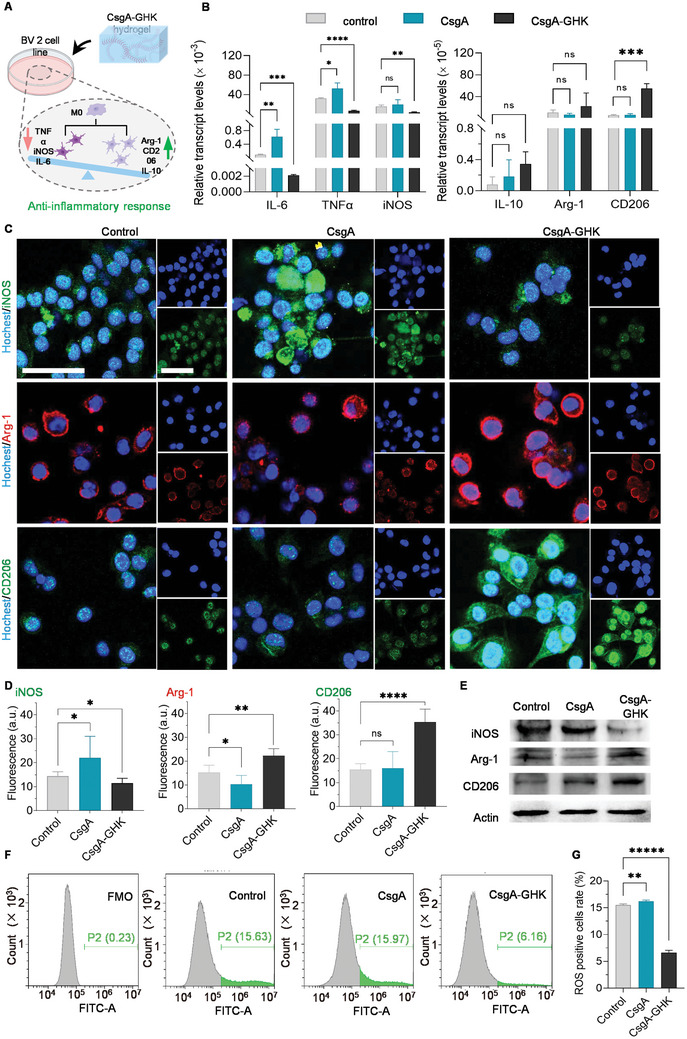
The analysis of microglia polarization. A) Schematic illustration of CsgA‐GHK hydrogel‐induced M2 microglial polarization with a down‐regulation of proinflammatory factors (IL‐6, TNF‐*α*, iNOS) and an up‐regulation of anti‐inflammatory factors (IL‐10, Arg‐1, CD206). B) The qRT‐PCR results showing the expression of proinflammatory factors (*IL‐6*, *TNF‐α* and *iNOS*) and anti‐inflammatory factors (*IL‐10*, *Arg‐1* and *CD206*) in BV2 cells following the treatment of CsgA or CsgA‐GHK hydrogels (*n* = 3). C) IF imaging showing the proportion of iNOS, Arg‐1 positive, and CD206 positive BV2 cells. Green/red fluorescence represents the M1/M2 microglia/macrophage phenotype marker iNOS or Arg‐1, CD206, respectively. Blue fluorescence represents the nuclear marker Hoechst 33342. Scale bars: 50 µm. D) Quantification of fluorescence intensity of iNOS, Arg‐1 and CD206 levels in each group (*n* = 5). E) WB results of iNOS, Arg‐1, and CD206 protein expression. F) The proportion of ROS‐positive BV2 cells analyzed by flow cytometry. FMO: fluorescence minus one. G) Quantification of flow cytometry results (*n* = 3). Data are presented as mean ± s.d. ns, *p* ≥ 0.05; *p < 0.05; **p < 0.01; ***p < 0.001; ****p < 0.0001; *****p < 0.00001.

As resident macrophages of the CNS, microglia are key immune effectors in inflammatory lesions and related neurological dysfunction.^[^ ^39^ ^]^ To evaluate the anti‐inflammatory properties of the CsgA‐GHK hydrogel, we cocultured a mouse microglia BV2 cell line with CsgA‐GHK hydrogel. The results of endotoxin detection (Figure S5, Supporting Information) and BV2 cell live/dead staining (Figure S6A, Supporting Information), along with the analysis of cell proliferation ability (Figure S6B, Supporting Information), verified that our hydrogels exhibit good biocompatibility. In addition, CsgA/CsgA‐GHK hydrogels exhibited extremely low swelling properties, which is beneficial for in vivo and in vitro experiments (Figure S7, Supporting Information). Microglia can exhibit pro‐inflammatory (M1) or anti‐inflammatory (M2) phenotypes that embrace specific markers (**Figure** **3**A). M1 markers primarily consist of inducible nitric oxide synthase (iNOS), interleukin‐6 (IL‐6), and tumor necrosis factor‐alpha (TNF‐*α*) while M2 markers include arginase‐1 (Arg‐1), cluster of differentiation 206 (CD206), and IL‐10.^[^ ^39^ ^]^ It was observed that both CsgA and CsgA‐GHK hydrogels significantly promoted the differentiation of BV2 cells, leading to a change in cell morphology from round to fusiform compared to the control group cultured in a cell culture dish (Figure S8A,B, Supporting Information). Gene expression analysis revealed that the mRNA levels of pro‐inflammatory cytokines (*IL‐6*, *TNF*, and *iNOS*) in BV2 cells cultured on the CsgA‐GHK hydrogel were significantly lower than those in the control groups. In contrast, cells cultured on the CsgA hydrogel exhibited significantly higher levels of these cytokines, except for iNOS. The mRNA expression levels of anti‐inflammatory cytokines (*IL‐10*, *Arg‐1*, and *CD206*) were higher in the CsgA‐GHK group than in the control group, with *CD206* expression being significantly upregulated. In contrast, cells cultured on the CsgA hydrogel did not show obvious changes compared to the control group (Figure 3B).

Immunofluorescence (IF) imaging of BV2 cells indicated a significant decrease in the number of iNOS‐positive cells and a significant increase in Arg‐1/CD206‐positive cells in the CsgA‐GHK group compared to the control group (Figure 3C,D). The expression pattern of marker proteins in the CsgA group was opposite to that in the CsgA‐GHK group, consistent with the qRT‐PCR results. Evaluation of inflammation regulation, including Arg‐1, iNOS, and CD206 protein expression, through WB analysis demonstrated that the CsgA‐GHK hydrogel promotes M2 microglial polarization (Figure 3E).

We also examined ROS levels in BV2 cells cultured with CsgA and CsgA‐GHK hydrogels, as M1‐polarized microglia are known to secrete high levels of ROS, a key component of the inflammatory response.^[^ ^3^, ^32^ ^]^ Fluorescence imaging revealed that the proportion of ROS‐positive cells in the CsgA‐GHK group was approximately 10%, representing a 50% reduction compared to the control and CsgA group, where both groups exhibited around 20% ROS‐positive cells (Figure S8C,D, Supporting Information). Furthermore, we quantified the imaging results using flow cytometry, which showed that the proportion of ROS‐positive cells in the CsgA‐GHK group was 6%, indicating a 60% reduction compared to the control and CsgA group, where both groups had approximately 15% ROS‐positive cells (Figure 3F,G). These results collectively suggest that the CsgA‐GHK gel significantly decreased M1 microglial polarization and promoted M2 microglial polarization, which in turn may aid in SCI repair and nerve regeneration.

### 2.3 Determination of the Inflammatory Response Pathway Activated by CsgA‐GHK Hydrogel

**Figure 4:**
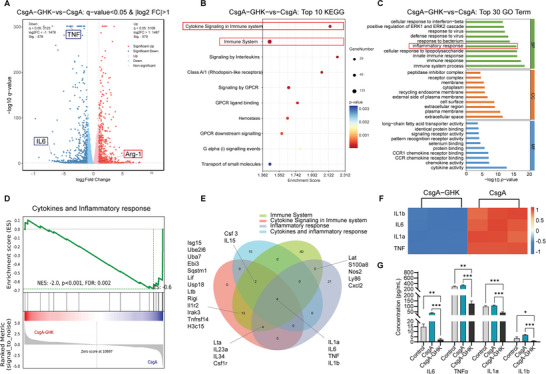
The RNA‐seq of BV2 cells treated with CsgA‐GHK and CsgA hydrogels. A) The volcano plots of identified up‐regulated and downregulated genes. B) The KEGG pathway classification of significant differential genes (*p* < 0.05, fold change > 2). The red boxes mark the signal pathways of interest. C) The GO pathway enrichment analysis of significant differential genes (*p* < 0.05, fold change > 2). The red box marks the signal pathway of interest. D) The GSEA enrichment analysis of cytokines and inflammatory response pathway (*p* = 0.003). E) The Venn graph of the four selected signal pathways of CsgA‐GHK versus CsgA and CsgA‐GHK versus control groups. F) The heat map of *IL1b*, *IL6*, *IL1a* and *TNF* genes expressions. G) ELISA analyses of IL6, TNF, IL1a and IL1b proteins (*n* = 3). Data are presented as mean ± s.d. *p < 0.05; **p < 0.01; ***p < 0.001.

To further elucidate the molecular mechanisms of the effects of CsgA‐GHK hydrogel treatment on BV2 cells, RNA‐seq data analysis was performed. Three sample groups were analyzed: cells that were cultured on CsgA/CsgA‐GHK hydrogels and without gel as the control, each with three biological replicates. Principal Component Analysis of the RNA‐seq data showed that the variance in CsgA, CsgA‐GHK and control groups was 87.24%, indicating distinct gene expression patterns (Figure S9A, Supporting Information). The variance within the CsgA‐GHK samples was 4.32%, and that within CsgA samples was 2.61%, the control samples was 5.39%, demonstrating consistency within each group. Correlation coefficient analysis between samples showed high values (94–99%), indicating good repeatability and reliability of the experimental setup for subsequent bioinformatics analysis (Figure S9B, Supporting Information). In comparison to the control group, CsgA‐GHK hydrogel treatment led to the upregulation of 257 genes and downregulation of 332 genes (Figure S10A, Supporting Information, *p* < 0.05, fold change > 2), highlighting the impact of the hydrogel on gene expression profiles. When compared to the CsgA group, CsgA‐GHK hydrogel treatment led to the upregulation of 579 genes and downregulation of 578 genes (**Figure** **4**A and *p* < 0.05, fold change > 2), further indicating the influence of the GHK peptide on gene expression profiles. Key genes such as *IL6*, *TNF*, and *Arg‐1* showed differential changes in expression levels, consistent with qRT‐PCR results (Figure 3B), validating the accuracy of the RNA‐seq data.

The Kyoto Encyclopedia of Genes and Genomes (KEGG) pathway analysis revealed that the immune system was the most significantly enriched pathway. Also, another pathway “the cytokine signaling in immune system” had been enriched, indicating the impact of CsgA‐GHK hydrogel treatment on immune‐related processes (Figure 4B and Figure S10B, Supporting Information). Gene Ontology (GO) pathway analysis also revealed enrichment in the inflammatory response pathway in biological processes (BP). Molecular functional (MF) classifications highlighted pathways related to immune inflammatory responses, such as immune response, and CCR chemokine receptor binding signaling pathways (Figure 4C and Figure S10C, Supporting Information). These are related to attracting monocytes/macrophages to the site of inflammation.^[^ ^40^ ^]^ Moreover, Gene Set Enrichment Analysis (GSEA) results showed significant changes in cytokines and inflammatory response pathway (Figure 4D and Figure S10D, Supporting Information). Therefore, the CsgA‐GHK hydrogel induced changes in inflammatory signaling pathways when compared to both the control and CsgA groups. The four screened signaling pathways (i.e., the immune system, the cytokine signaling in immune system, inflammatory response, cytokines and inflammatory response) were analyzed respectively by Venn diagrams to identify the correlations of CsgA‐GHK versus CsgA and CsgA‐GHK versus control groups, showing that there are 68, 23, 34 and 21 genes in common across these pathways, respectively (Figure S10E, Supporting Information). Additionally, a Venn diagram of these common genes showed that *IL1b*, *IL6*, *IL1a*, and *TNF* genes were contained in all screened pathways (Figure 4E). The heat map of gene expressions demonstrated that CsgA‐GHK hydrogel treatment downregulated the expression of several pro‐inflammatory factors compared to both the control and CsgA groups (Figure 4F and Figure S10F, Supporting Information), which were verified by RT‐qPCR (Figure 3B and Figure S9G, Supporting Information). The ELISA results also confirmed that CsgA‐GHK hydrogel has a regulatory effect on the expression IL6, TNF, IL1a and IL1b proteins, when compared to CsgA or control group (Figure 4G). Overall, these findings suggest that the CsgA‐GHK hydrogel activates the inflammatory response pathway to exert its anti‐inflammatory function by regulating the expression of proinflammatory cytokines.

### 2.4 Study of NSCs Differentiation on CsgA‐GHK Hydrogel

NSCs are versatile, self‐renewing cells capable of differentiating into diverse types of neural cells and glial cells, crucial for the establishment of neural networks.^[^ ^41^ ^]^ For nerve injury repair, inflammation can expedite the differentiation of NSCs into glial cells, a process that may not be conducive to effective injury repair.^[^ ^42^ ^]^ Therefore, the observed anti‐inflammatory effects of CsgA‐GHK hydrogel prompted us to further investigate its role in the differentiation process of NSCs. For this purpose, NSCs were cultured with CsgA or CsgA‐GHK hydrogels, and their proliferation and differentiation of neurons and glial cells were analyzed at different time points (Figure S11, Supporting Information). Initially, we assessed the biocompatibility of CsgA and CsgA‐GHK hydrogels with NSCs and neurons. Live/dead staining and a CCK‐8 assay were conducted on NSCs and a neuronal cell line PC‐12 cells, revealing that cells cultured on CsgA or CsgA‐GHK hydrogels exhibited similar proliferative activity to the control group (Figure S11B,C, Supporting Information). Moreover, the hydrogels demonstrated the ability to maintain the stemness of NSCs during the early stages of culture (Figure S11B, Supporting Information).

**Figure 5:**
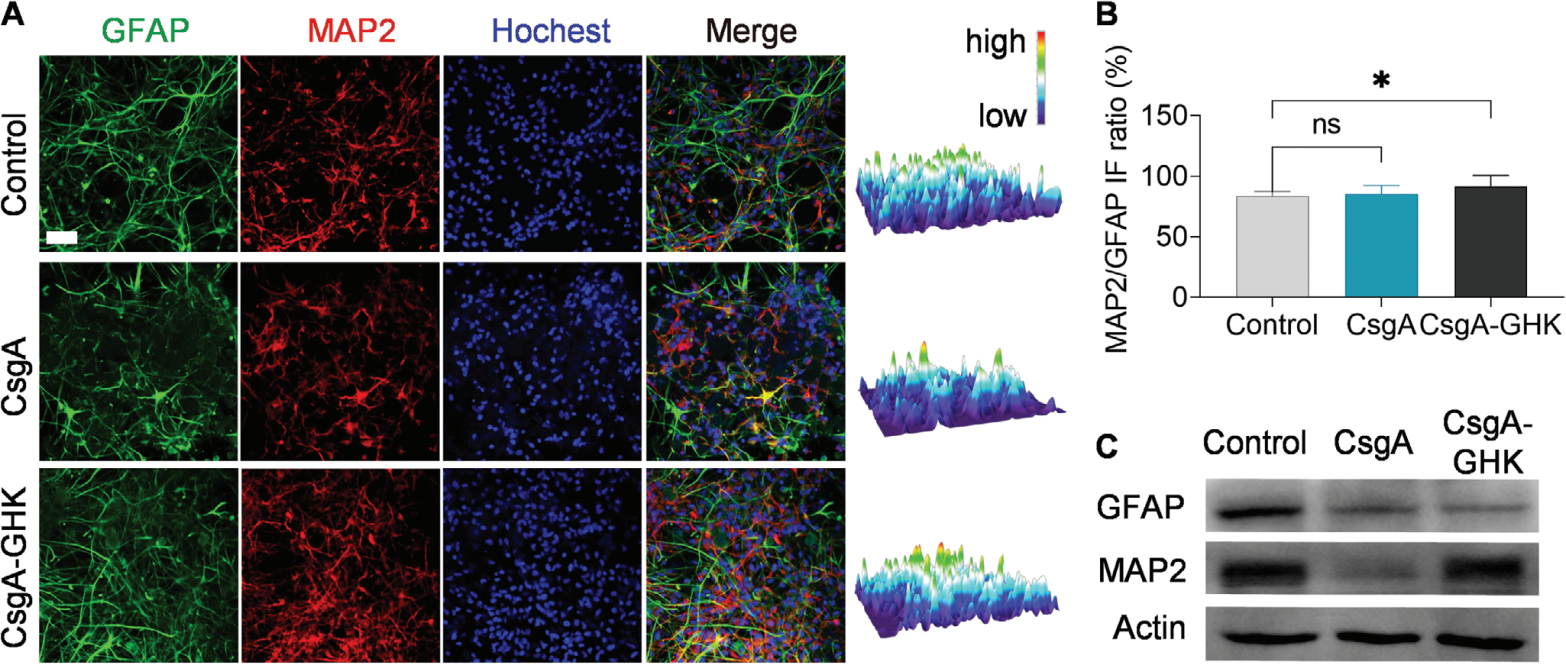
Comparison of the NSC differentiation behavior. A) IF imaging of NSC neuron and astrocyte differentiation after 5 days on control or hydrogel conditions. Red, green and blue IF represents neuron marker MAP2, astrocyte marker GFAP, and nuclear marker hochest, respectively. The rightmost panel shows the 3D heat map of MAP2. Scale bars: 50 µm. B) Comparison of MAP2/GFAP IF ratios among the three groups (*n* = 6). C) WB analysis of GFAP and MAP2 protein expression in NSCs after 5 d culture. Data are presented as mean ± s.d. ns, *p* ≥ 0.05; *p < 0.05.

Compared to the control group, CsgA and CsgA‐GHK hydrogels promoted the gene expression of the neuronal differentiation marker microtubule‐associated protein‐2 (*MAP2*), while inhibiting the expression of the gliocyte marker glial fibrillary acidic protein (*GFAP*) gene under these culture conditions (Figure S11D, Supporting Information). Additionally, NSCs cultured on the hydrogels exhibited higher levels of *MAP2* and lower levels of *GFAP* expression compared to the control group, although the expression of *GFAP* did not show a significant difference between the CsgA and CsgA‐GHK groups. Furthermore, we analyzed the protein expressions of MAP2 and GFAP using IF and WB. The 3D heat maps of MAP2 IF images and WB results both indicated that MAP2 was highly expressed in the control and CsgA‐GHK groups compared to the CsgA group (**Figure** **5**A,C). The expression of GFAP protein significantly decreased in the CsgA and CsgA‐GHK groups compared to the control group, according to the IF image 3D heat maps and WB results (Figure 5C and Figure S11E, Supporting Information). The ratio of fluorescence intensity of MAP2/GFAP in the control, CsgA, and CsgA‐GHK groups were 83.7%, 85.4%, and 91.4%, respectively, with the CsgA‐GHK group showing significantly higher values than the other groups (Figure 5B). However, the expression trend of MAP2 protein levels did not entirely align with that of gene expression levels in the results, likely due to differences in transcription levels and protein accumulation. Nevertheless, these results clearly demonstrated that the CsgA‐GHK hydrogel could promote neuronal differentiation of NSCs, as evidenced by the upregulation of MAP2 and downregulation of GFAP. Oxidative damage from aging or nerve injury inhibits neural stem cells differentiate into neurons, leading to increased glial cell differentiation, which is not conducive to nerve regeneration.^[^ ^43^ ^]^ The antioxidant and anti‐inflammatory capabilities of CsgA‐GHK hydrogel have been thoroughly demonstrated in the results presented above. Therefore, we propose that the CsgA‐GHK hydrogel creates a favorable microenvironment for neuronal growth while reducing glial cell differentiation, resulting in altered GFAP and MAP2 expression.

### 2.5 Evaluation of the CsgA‐GHK Promoted Functional Recovery of Rats after SCI

**Figure 6:**
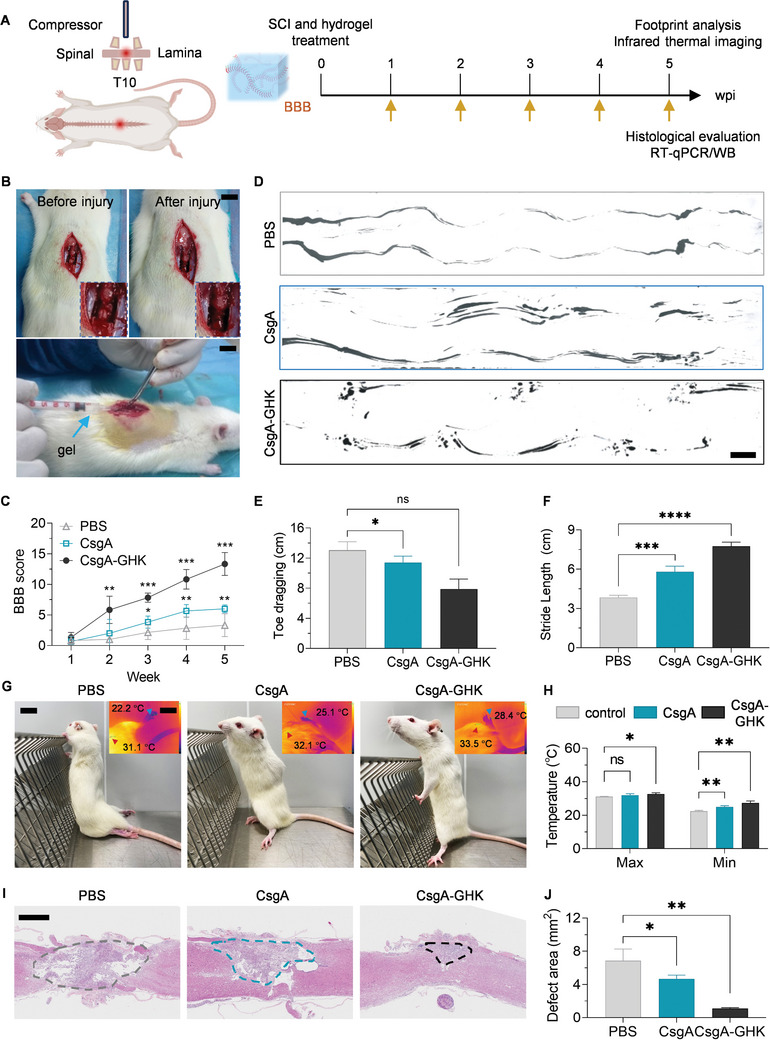
Evaluation of the functional recovery of rats after SCI. A) The experimental timeline schematics. B) Pictures showing the establishment of the spinal cord compression injury model and the subsequent hydrogel implantation. Scale bars: 1 cm. C) Comparison of the open field locomotion‐BBB hindlimb score after treatments (*n* = 6). D) Representative footprints of rats treated with different materials. E) Toe dragging and F) stride length used to quantify the recovery of locomotion 5 weeks after injury (*n* = 6). G) Representative images of toes and feet of rats standing on table with forelimbs grip the upright 90 °C net frame. Insets in each group show the infrared measurements of the body and hind limb fingertip temperature of rats. Scale bars: 2 cm. H) Statistics of maximum and minimum temperatures in the three groups (*n* = 3). I) HE staining showing the morphology of transverse spinal cord sections after treatments. Scale bars: 1 mm. J) Damage cavity area statistics of HE staining results (*n* = 3). Data are presented as mean ± s.d. ns, *p* ≥ 0.05; *p < 0.05; **p < 0.01; ***p < 0.001; ****p < 0.0001.

Building on the results in the previous experiments, where the CsgA‐GHK hydrogel demonstrated suitable mechanical properties, exceptional anti‐inflammatory characteristics, and the ability of promoting NSCs to differentiate into neurons, we proceeded to evaluate the application efficacy of the hydrogel in a SCI rat model. The experimental timeline schematic was depicted in **Figure** **6**A. The degradability test results, both in vivo and in vitro, illustrated that the CsgA‐GHK hydrogel underwent gradual degradation over one month (Figure S12, Supporting Information). To assess rat functional recovery following SCI, the Basso Beattie Bresnahan (BBB) motor assessment was employed. The surgical procedure was outlined in Figure 6B. Immediately post‐spinal cord compression injury, the animals displayed complete paralysis (score of 0) in both hindlimbs (Figure 6C). Five weeks after SCI, the hindlimb BBB scores did not exceed 5 in the control group, indicating limited self‐healing capacity. In contrast, the CsgA‐GHK group exhibited statistically significant locomotor functional recovery within the first 2 weeks post‐operation (Figure 6C). In the CsgA hydrogel group, locomotor function was comparable to that in the PBS group at 2 weeks post‐injury but showed significant improvement by the third week after SCI, albeit to a much lesser extent than the CsgA‐GHK hydrogel (Figure 6C). These results indicated that the functional hydrogel could enhance rat movement recovery. By the fifth week post‐operation, most rats implanted with CsgA‐GHK hydrogel exhibited normal weight support (score of 13), while those treated with the CsgA hydrogel displayed plantar placement with support (score of 5), and rats in the PBS group demonstrated extensive ankle movement (score of 3) (Figure 6D–F). These outcomes strongly suggest that the CsgA‐GHK hydrogel significantly enhanced functional recovery following SCI.

Following SCI, hind legs are paralyzed, blood circulation is slowed, and temperature regulation is affected. The hind limb and body temperatures of rats were measured using an infrared thermal imager, revealing an increase in body temperature after hydrogel treatment, particularly a significant elevation in hindfoot temperature after CsgA‐GHK hydrogel application compared to the PBS and CsgA groups (Figure 6G,H). Similarly, normal spinal cord morphology, which is typically disrupted due to the loss of normal nervous tissue at the injury site, was observed in tissue hematoxylin and eosin (HE) staining (Figure 6I). In longitudinal spinal cord sections, the cavitary area averaged 6.87 mm^2^ in the PBS group and 4.6 mm^2^ in the CsgA group. In contrast, the CsgA‐GHK hydrogel reduced vacuolation and the cavitary area in the CsgA‐GHK group (average of 1.1 mm^2^) at 5 weeks post‐surgery (Figure 6J).

### 2.6 Evaluation of the Inflammation Suppression Property of CsgA‐GHK Hydrogel in Rats after SCI

**Figure 7:**
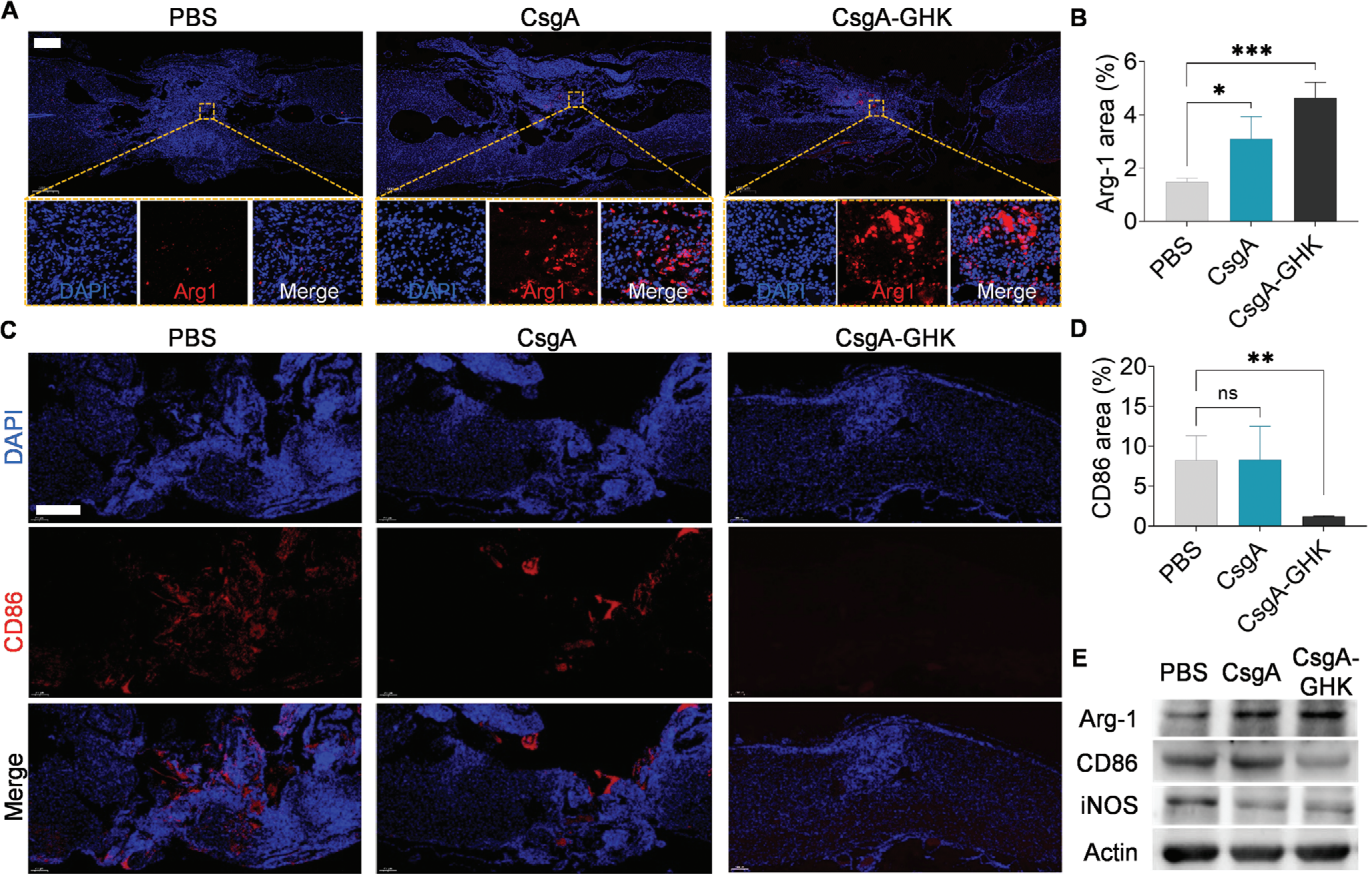
Evaluation of inflammation suppression after SCI. A) IF images comparing the Arg‐1 density in Arg‐1‐positive cells. Red IF represents Arg‐1 and blue IF represents DAPI for stained nucleus. Scale bars: 500 µm; 50 µm in the zoomed‐in images. B) The quantified fluorescence intensity of Arg‐1 (*n* = 3). C) IF images comparing the CD86 density in CD86‐positive cells. Red IF represents CD86 and blue IF represents DAPI for stained nucleus. Scale bars: 200 µm. D) The quantified fluorescence intensity of CD86 (*n* = 3). E) WB analysis of CD68, iNOS, and Arg‐1 proteins. Data are presented as mean ± s.d. ns, *p* ≥ 0.05; *p < 0.05; **p < 0.01; ***p < 0.001.

Inflammatory reactions often trigger a cascade of secondary insults following SCI. Therefore, suppressing inflammation can create a conducive environment for SCI repair. The CsgA‐GHK gel's ability to promote M2 polarization of microglia and reduce ROS was validated in our in vitro experiments. Subsequently, the spinal cords of rats from each treatment group were collected to assess the inflammation resulting from the injury and materials used. The density of Arg‐1‐positive cells was notably higher at the injury site in the hydrogel treatment groups compared to the PBS group (**Figure** **7**A). Particularly in the CsgA‐GHK group, the Arg‐1 fluorescence area was significantly larger than that of the PBS group (Figure 7B). Moreover, the immunofluorescence (IF) intensity of proinflammatory factor CD86 increased in the PBS and CsgA groups at the center of the injury site, surpassing that in the CsgA‐GHK group (Figure 7C,D). The WB results further confirmed that transplantation of the CsgA‐GHK hydrogel reduced the expression of the proinflammatory factors CD86 and iNOS proteins while elevating the levels of the anti‐inflammatory factor Arg‐1 (Figure 7E). These results indicated the important role of GHK peptide that can suppress inflammation in the early stages and effectively mitigate long‐term chronic inflammation, and thus underscore the immunomodulatory properties of the CsgA‐GHK hydrogel.

It is worth noting that GHK as a copper‐binding tripeptide has been widely used for wound healing and antiaging skincare.^[^ ^44^ ^]^ However, our study has unveiled a new dimension to the potential of the GHK tripeptide by showcasing its efficacy in the realm of nerve injury repair. Specifically, our hydrogel system, formed through the fusion expression of GHK and the CsgA self‐assembly protein, significantly reduces the amount of GHK while enhancing its stability and enabling long‐term slow release at the injury site, thereby improving the inflammatory microenvironment. Microglia‐coordinated inflammatory responses often have destructive effects on all major cell types in the central nervous system.^[^ ^39^ ^]^ Activated microglia can undergo heterogeneous differentiation, resulting in proinflammatory (M1‐phenotype) and anti‐inflammatory (M2‐phenotype) microglia.^[^ ^45^ ^]^ M1‐phenotype microglia release cytokines such as IL‐1α, IL‐1β, and TNF, which promote the transformation of astrocytes into highly reactive, neurotoxic cells, and produce reactive oxygen species (ROS) that lead to excitotoxicity.^[^ ^46^, ^47^ ^]^ In our in vitro experiments, we confirmed that the CsgA‐GHK hydrogel significantly down‐regulated the cytokines TNF‐α, IL‐6, IL‐1α, and IL‐1β in BV2 cells. Previous studies have shown that metal ion homeostasis is disrupted following spinal cord injury, with Fe^2^⁺/Cu^2^⁺ in cells generating excessive ROS through the Fenton reaction.^[^ ^48^, ^49^ ^]^ The CsgA‐GHK gel was found to reduce ROS levels in vitro and exhibited excellent anti‐inflammatory properties in vivo, likely due to the GHK peptide's ability to bind copper. Consequently, the expression of M1‐phenotype microglia marker proteins (iNOS, CD86) was significantly down‐regulated, while M2‐phenotype microglia marker proteins (CD206, Arg‐1) were significantly up‐regulated following treatment with the CsgA‐GHK gel. This evidence supports the conclusion that our CsgA‐GHK hydrogel possesses remarkable capabilities to modulate the oxidative microenvironment of cells.

### 2.7 Assessment of CsgA‐GHK Hydrogel‐Triggered Neuronal Regeneration after SCI

**Figure 8:**
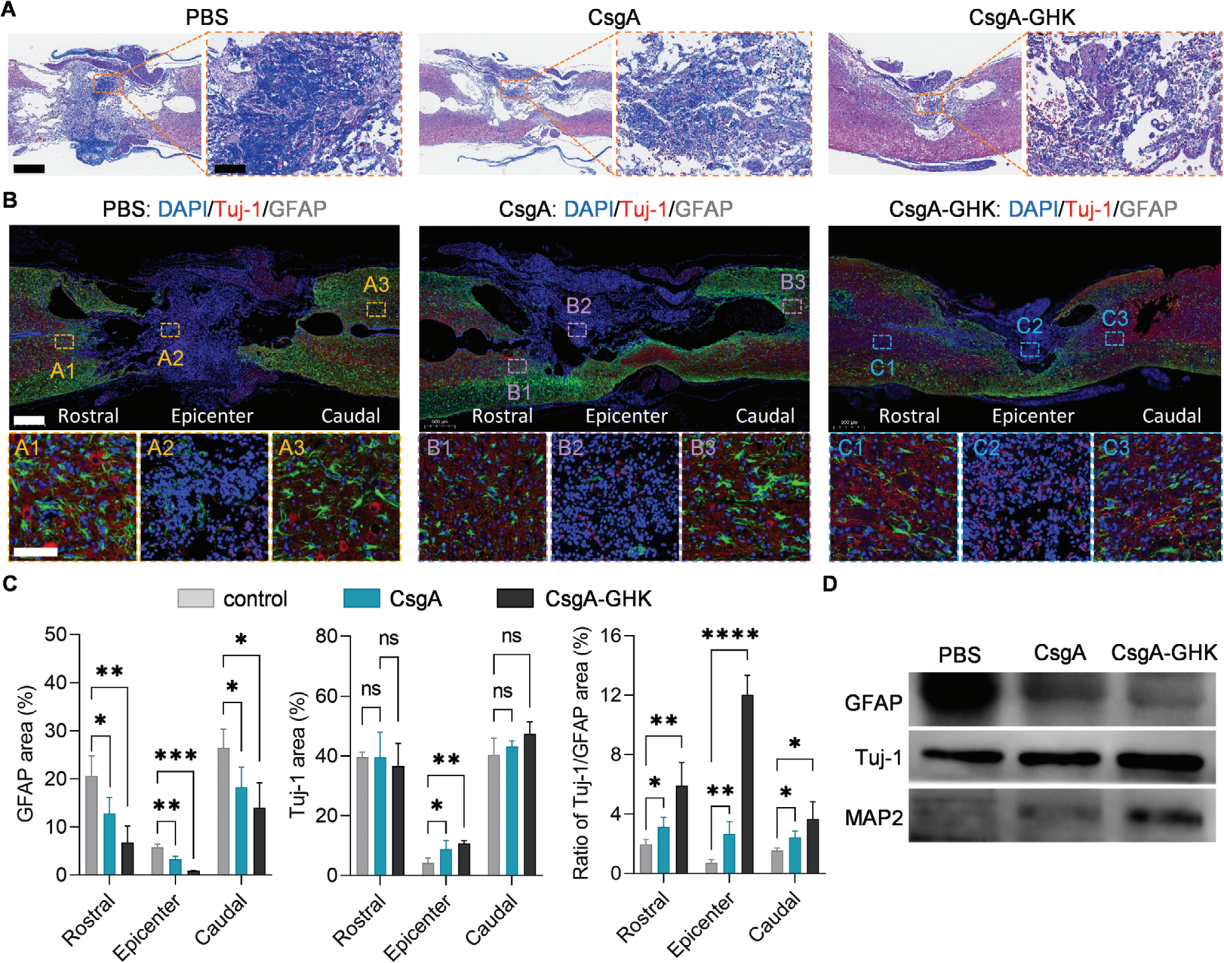
Regeneration of neurons after SCI. A) The Masson staining images of spinal cord sections after the treatments. The enlarged area shows collagen staining. Scale bars: 500 µm; 100 µm in the zoomed‐in images. B) IF imaging of Tuj‐1 and GFAP in different parts of the injured spinal cord. Red, green and blue IF represent the neuronal marker Tuj‐1, the astrocytic marker GFAP and DAPI for stained nucleus, respectively. The fluorescence was measured at three areas of interest: the rostral and caudal borders, and the injury site center. Scale bars: 500 µm in top panel; 100 µm in bottom panel. C) Quantification of the IF intensity of GFAP and Tuj‐1 IF. The IF intensity ratio between the two markers was also plotted for comparison (*n* = 3). D) WB analysis of the neuronal and astrocytic iconic protein markers at the injury site center of the spinal cord. Data are presented as mean ± s.d. ns, *p* ≥ 0.05; *p < 0.05; **p < 0.01; ***p < 0.001; ****p < 0.0001; *****p < 0.00001.

To further investigate the mechanisms underlying functional recovery after SCI following CsgA‐GHK hydrogel treatment, the histological changes of spinal cord tissue were analyzed to assess neural regeneration and astroglial scar formation at the injury site. Initially, distinct collagen deposition was observed in spinal cord lesions post‐SCI using Masson's trichrome staining in the PBS group. Compared to the PBS group, hydrogel implantation led to a reduction in collagen content, with the CsgA‐GHK group exhibiting minimal collagen accumulation at SCI sites (**Figure** **8**A). Subsequently, the expressions of neuronal marker Tuj‐1 and the astrocytic marker GFAP were detected using immunofluorescence. In the CsgA and CsgA‐GHK hydrogel groups, Tuj‐1 protein was highly expressed at the center of the lesion area, whereas its expression was relatively scarce in the PBS group (Figure 8B). However, the expression of Tuj‐1 protein did not significantly differ among the PBS, CsgA, and CsgA‐GHK groups at the rostral and caudal borders. These observations suggest that CsgA and CsgA‐GHK hydrogels facilitated neuronal invasion into the center of the injury site (Figure 8C).

Furthermore, the expressions of GFAP protein were notably increased at the caudal and rostral regions, forming an astroglial scar around the lesion site in the PBS group, with more GFAP‐positive astrocytes compared to the CsgA and CsgA‐GHK hydrogel groups (Figure 8B). Consequently, CsgA and CsgA‐GHK hydrogels suppressed GFAP protein expression and astrocytic scar formation, creating a conducive microenvironment for subsequent axonal regeneration (Figure 8C). WB analysis aligned with the immunofluorescence results, the expression of two neuronal markers, Tuj1 and MAP2, in the CsgA‐GHK group was significantly higher than CsgA hydrogel and PBS group, while GFAP expression at the lesion site in the CsgA‐GHK group was markedly lower than in the CsgA and PBS groups (Figure 8D).

It has been established the mechanical properties of the extracellular matrix materials can influence the proliferation and differentiation of NSCs.^[^ ^50^ ^]^ NSCs exhibit distinct responses to varying stiffness levels, with poor survival observed in very soft (< 0.1 kPa) or very hard (>100 kPa) materials. NSCs tend to differentiate into neurons in reasonably soft materials (approximately 0.1–1 kPa) and into glial cells in slightly stiffer materials (around 7–10 kPa).^[^ ^51^ ^]^ In this study, the storage moduli of the CsgA/CsgA‐GHK hydrogels were 500 Pa, a stiffness level that aligns with the mechanical properties of spinal cord tissue (200 Pa).^[^ ^13^ ^]^ This similarity in mechanical properties suggests that the CsgA and CsgA‐GHK hydrogels may provide an environment conducive to neuronal regeneration while inhibiting astrocytic proliferation.

## 3 Conclusions

In summary, we report a single‐component hydrogel based on CsgA recombinant proteins for SCI treatment. The fusion of CsgA with GHK not only leverages the self‐assembling properties of CsgA but also harnesses the therapeutic potential of GHK, offering a modular approach for the preparation of active protein‐based biomaterials. CsgA‐GHK protein forms hydrogels through the entanglement of self‐assembling nanofibrils, precluding cumbersome preparation procedures or chemical crosslinkers that are common requisites in other systems. Importantly, CsgA‐GHK hydrogel exhibits mechanical properties that match those of spinal cord tissue, making it suitable for SCI treatment. The active module within the hydrogel demonstrates remarkable anti‐inflammatory properties. In vitro experiments show that the CsgA‐GHK hydrogel effectively promotes M2 microglial polarization, scavenges ROS, enhances neuronal differentiation of NSCs, and reduces glial cell differentiation. Furthermore, the hydrogel is successful in treating inflammation at the SCI lesion site, thereby improving the damaged microenvironment. This synergistic treatment approach significantly improved the functional regeneration of neurons, reduced the formation of scar tissue, and ultimately led to motor functional recovery in a SCI rat model. Considering the availability of multiple proteins with self‐assembling capabilities and the presence of diverse biologically active peptidic modules, we therefore anticipate that our work will raise the prospects of designing active protein‐based soft materials for advancing SCI treatment and other biomedical interventions.

## 4 Experimental Section

### Plasmid Construction

Target gene fragments (refer to Table S1, Supporting Information) were amplified using PCR with specific primers (refer to Table S2, Supporting Information) and *E. coli* genome template. The PCR‐amplified gene fragments and the plasmid pET‐22b^+^ were cleaved using Hind III and Xho I restriction enzymes (Thermo Scientific, USA) for 15 min at 37 °C. The cleaved gene fragments and plasmid were mixed and incubated with T4 DNA ligase (Invitrogen, USA) for 1 hour at 26 °C to facilitate ligation. The ligated DNA constructs were then transformed into BL21 (DE3) *E. coli* competent cells (Solarbio Life Sciences, China). Subsequently, the transformed cells were plated on LB media plates containing antibiotics to select the desired strains. All constructed plasmids were subjected to sequence verification by the Beijing Genomics Institute (BGI, China) to confirm the accuracy and integrity of the cloned gene fragments.

### Protein Expression and Purification

Bacterial seeds were cultured overnight in 40 mL LB at 37 °C and 200 rpm. The culture was then inoculated into 1 L LB at a ratio of 1:100 and cultured at 37 °C and 200 rpm. When the OD_600_ reached 0.8, isopropyl β‐D‐1‐thiogalactopyranoside (IPTG) was added to a final concentration of 0.5 mM. The culture was further incubated for 2 hours at 37 °C and 200 rpm to induce protein expression. The culture was centrifuged for 15 min at 4000 × *g* at 4 °C. Cell pellets (5 g) were lysed in 50 mL lysis buffer (8 m GdnHCl, 300 mm NaCl, 50 mm K_2_HPO_4_/KH_2_PO_4_, pH 7.2) for 24 h at 4 °C. Supernatants of the lysates were collected by centrifugation at 11000 × *g* for 1 h. The supernatants were loaded onto a His‐Select Ni NTA column (Sangon Biotech, China). The column was washed sequentially with KPI buffer (Buffer A: 300 mm NaCl, 50 mm K_2_HPO_4_/KH_2_PO_4_, pH 7.2) and 40 mm imidazole KPI buffer (Buffer B). Elution was performed using 300 mm imidazole KPI buffer (Buffer C) to release the target protein. The concentration of the recombinant protein was determined using a Nano Drop spectrophotometer (DeNovix, USA).

### Sodium Dodecyl Sulfate‐Polyacrylamide Gel Electrophoresis (SDS‐PAGE) and Coomassie Blue Staining

20 µL of freshly eluted protein solutions were mixed with 5 µL of 5× SDS loading buffer (Beyotime, China). 10 µL of the mixed solutions were loaded into the lanes of an SDS‐PAGE gel along with a standard protein ladder (Solarbio Life Sciences, China). The gel was run at 90 V for 90 min using an electrophoresis apparatus (Tanon, China) in SDS running buffer (Beyotime, China). The gel was stained with Coomassie Blue solution (Beyotime, China) for 1 h. Subsequently, the gel was destained with ddH_2_O for 1 h twice to remove excess stain. The gels were imaged using a Tanon Gel Image System to visualize the separated protein bands.

### Circular Dichroism (CD) Analysis

The recombinant protein, with a concentration of 0.2 mg mL^−1^ in ddH_2_O, was subjected to CD analysis using a Chirascan Plus spectrometer (Applied Photophysics Ltd, UK).

### Thioflavin T (ThT) Assay

To study the aggregation kinetics of the recombinant protein nanofiber, the fresh recombinant protein solutions (5 mg mL^−1^) in buffer C at pH 7.2 were prepared. ThT at a concentration of 1 × 10^−2^ mm (Med Chem Express, USA) was added to the solutions. The protein solutions with ThT were transferred into 96‐well plates for analysis. ThT fluorescence at 490 nm was recorded at regular intervals of every 3 h for a total duration of 30 h. The Varioskan LUX spectrophotometer (Thermo Fisher, USA) was used to measure the fluorescence intensity.

### Congo Red (CR) Binding Assay

The fresh recombinant protein solutions (5 mg mL^−1^) in buffer C at pH 7.2 were prepared. 1.4 × 10^−1^ mm Congo Red solution (Solarbio Life Sciences, China) was added to these solutions. The absorption spectra were recorded in the range from 375 to 700 nm using the spectrophotometer (Varioskan LUX, Thermo Fisher, USA).

### Hydrogel Samples Preparation

The 1 mL of fresh recombinant protein at a concentration of 5 mg mL^−1^ in Buffer C was placed in a 1.5 mL EP tube and allowed to form gel at room temperature (20 °C ± 2) for more than 48 h. The mixture was then centrifuged at 10 000 rpm for 5 min to remove excess water, yielding approximately 500 µL of hydrogel, which was further soaked with 500 µL ddH_2_O (3 times) and 500µL PBS (3 times) for 12 h each time at room temperature to remove the residual imidazole. During cell experiments, the hydrogel was transferred using a pipette, while a syringe was used for animal experiments.

### Transmission Electron Microscopy (TEM)

10 µL sample was deposited on a carbon‐coated copper grid and allowed to sit for 10 min. The excess sample was carefully removed using filter paper to ensure a thin and uniform layer on the grid. A negative stain was performed by adding 10 µL of 2% (w/v) phosphotungstic acid (Solarbio Life Sciences, China) to the grid for 1 min. The excess stain was removed, and the sample was washed three times with ddH_2_O before and after staining to remove any residual stain. The prepared grid was loaded into a Talos F200S G2 transmission electron microscope (FEI NanoPorts, Czech Republic) for imaging.

### Scanning Electron Microscopy (SEM)

Hydrogel samples were subjected to a temperature gradient by freezing at −20 °C and −80 °C for 24 h. To preserve the original internal crosslinked structure, the samples were flash‐frozen using liquid nitrogen. The flash‐frozen hydrogel samples were sputter coated with platinum (Pt) using a high vacuum ion sputtering instrument EM ACE600 (Laica, Germany) for 80 s. The platinum‐coated samples were scanned using a Hitachi SU8010 field‐emission scanning electron microscope (Hitachi, Japan) at an acceleration voltage of 5 kV. Images were acquired using an in‐lens secondary electron (SE) detector.

### Rheological Measurement

The rheological characterization of the samples involved the following experiments using an 8 mm parallel‐plate geometry with a gap size of 800 µm on a DHR‐2 rheometer (TA, USA). Samples (100 µL) were allowed to equilibrate for 3 min before each measurement. Strain sweep tests were carried out with strains ranging from 0.1% to 100% at 37 °C and a fixed frequency of 1 Hz. Frequence sweep tests were carried out with frequencies ranging from 0.01 to 10 Hz at 37 °C and a fixed strain of 10%. Time sweep tests were carried out with two fixed strains (γ) of 1% and 100% at 37 °C and a fixed frequency of 1 Hz.

### Endotoxin Test

The endotoxin level of CsgA/CsgA‐GHK hydrogel was tested by an endotoxin test kit (Beyotime, China). According to kit instruction, 10µL hydrogel samples that used for cell experiments were added into the reaction system and observed any color changes to analyze the endotoxin content.

### Cell Lines and Cell Culture

Mouse nerve microglia BV2 and neuron PC‐12 cell lines were purchased from Oricell and Pricella, respectively. BV2 cells were cultured in MEM (Gibco, USA), while PC‐12 cells were cultured in 1640 medium (Gibco, USA), both supplemented with 5% fetal bovine serum (Gibco, USA). Neural stem cells (NSC) were extracted from fetal rat brain tissue and cultured in rat neural stem cell culture medium (OriCell, China). 10 µL of hydrogel was absorbed at the bottom of a 96/24‐well plate. The gun tip was inverted to evenly spread the hydrogel across the well. Subsequently, the advisable cells and medium were added to the hydrogel‐coated wells. The cells were cultured at 37 °C with 5% CO_2_ in a cell incubator (Memmert, Germany).

### Cell Counting Kit‐8 (CCK‐8) Assay

After culturing cells on hydrogels for 24 h in a 96‐well plate, 10 µL of CCK‐8 solution (Med Chem Express, USA) was added to each well containing 100 µL of medium. The culture plate was then placed in the incubator for 1 hour to allow the CCK‐8 solution to interact with the cells. The absorbance at 450 nm was measured using an EPOCH2 microplate reader (Bio Tek, USA).

### Live/Dead Cell Staining Assay

Following the instructions of the Calcein/PI Cell Viability/Cytotoxicity Assay Kit (Beyotime, China), the two reagents from the kit were mixed and diluted to a 2 × concentration in the cell culture medium. After culturing cells on hydrogels for 24 hours in a 96‐well plate, 100 µL of the 2 × Calcein/PI mix medium was added to each well containing 100 µL of medium. The culture plate was then placed in the incubator for 30 min to allow the staining solution to interact with the cells. Finally, the cells were observed under a fluorescence microscope Vert. A1 (ZEISS, Germany).

### Quantitative Reverse Transcription Polymerase Chain Reaction (qRT‐PCR)

Total RNA from the cells was extracted using TRIzol reagent (Invitrogen, USA), in combination with the corresponding RNA extraction kit (Omega, USA). Reverse transcription of the extracted RNA was carried out using the Prime Script RT reagent Kit with gDNA Eraser (Takara, Japan). qRT‐PCR was performed using the TB Green Premix Ex Taq II FAST qPCR kit (akara, Japan). The qPCR analysis was conducted on the qTOWER RT‐PCR system (Roche, Germany). The primers used for qRT‐PCR analysis are listed in Table S2 (Supporting Information).

### Immunofluorescence (IF)

Cells were fixed in 4% paraformaldehyde for 15 min to preserve cellular structures. Permeabilization was carried out using 0.5% Triton X‐100 for 15 minutes. Cells were washed three times with PBS containing 5% Tween (PBST). Blocking was performed using a solution of 3% BSA and 10% sheep serum in PBS at 37 °C for 1 h. Then, cells were incubated with primary antibodies (diluted 1:200) in the blocking solution for 1.5 h. After washing four times with PBST, cells were incubated for 1 h with secondary antibodies (1:500; Beyotime, China). After another four washes in PBST, cellular DNA was stained with DAPI (1:500; Beyotime, China). The stained cells were observed under a confocal microscope (Nikon, Japan). Image analysis was performed using the software Image J. The primary antibodies information in the method “Western blotting.”

### Reactive Oxygen Species (ROS) Assay

The final concentration of DCFH‐DA was 10 µmol L^−1^ after dilution of 1:1000 with serum‐free medium (CA1410, Beyotime, China). Remove the cell culture medium and add an appropriate volume of diluted DCFH‐DA. Incubate in a cell incubator at 37 °C for 1h. The cells were washed three times with serum‐free cell culture solution, and then observed under the Vert. A1 fluorescence microscope (ZEISS, Germany) or collected for analysis using the CytoFLEX flow cytometer (Beckman Coulter, USA).

### Western Blotting (WB)

The samples were lysed using RIPA lysis buffer (Beyotime, China). The total protein concentration was determined using a BCA Protein Assay Kit (Beyotime, China). After SDS‐PAGE (Tanon, China), the proteins were transferred onto a hydrophilic polyvinylidene fluoride capsule (Roche, Germany) by the bio‐rad Trans‐Blot and incubated with indicated primary antibodies. Then, the capsule was further incubated with horseradish peroxidase‐labeled secondary antibodies (1:2000, Beyotime, China). The blots were visualized using Clarity Western ECL Substrate (Bio‐Rad, USA). The primary antibodies used in this research were anti‐Actin (1:2000, AA128, Beyotime, China), anti‐iNOS (1:1000, 18985‐1‐AP, Proteintech, China), anti‐Arg‐1 (1:1000, 66129‐1‐Ig, Proteintech, China), anti‐CD206 (1:1000, JF0953, Invitrogen, USA), anti‐MAP2 (1:1000, ab11267, abcam, UK), anti‐GFAP (1:1000, PA5‐16291, Invitrogen, USA), anti‐Tuj1 (1:1000, ab78078, abcam, UK), and anti‐CD86 (1:1000, sc‐28347, Santa Cruz Biotechnology, USA).

### ELISA Analysis

The BV2 cells were cultured on CsgA/CsgA‐GHK hydrogel for 3 d, then the cell culture medium was obtained for IL6, TNFα, IL1a and IL1b protein concentrations detection (solarbio, China).

### Transcriptometric and Bioinformatics Analysis

The RNA libraries were sequenced by OE Biotech, Inc (Shanghai, China). Bioinformatic analysis was performed using the OECloud tools at https://cloud.oebiotech.com/task/. The volcano map (or other graphics) was drawn based on the R (https://www.r‐project.org/) on the OECloud platform (https://cloud.oebiotech.com/task/).

### Rat SCI Model

Adult female Sprague‐Dawley rats were purchased from the Experimental Animal Center of Zhejiang Province. The procedures and treatment were approved by the Experimental Animal Ethics Committee of Wenzhou Institute, University of Chinese Academy of Sciences (Issue No. WIUCAS21101838). Rats were anesthetized by intraperitoneal injection of 3% phenobarbital (0.1 mL/100 g). Then T10 laminectomy was performed to expose the spinal cord. The spinal cord was compressed using an obtuse forcep (F12018‐14, Rayward, China) with a closing force of 500G for 30 s. Successful SCI model establishment was confirmed by observing specific signs, i.e., swinging and sudden drooping of the tail, the spinal cord is swollen and congested. After the injury, 5 µL of CsgA or CsgA‐GHK hydrogel was injected into the SCI site for the treatment groups. The SCI model group received PBS as the control treatment. Muscle, fascia, and skin were sutured layer by layer after the compression. Bladder management included manual emptying twice daily until urinary function was restored. Rats were randomly assigned to three groups (*n* = 6 each): PBS, CsgA, and CsgA‐GHK.

### Behavioral Assessment

Basso Beattie Bresnahan (BBB) motor assessment was performed weekly from the start of surgery until week 5 to assess hind limb mobility after SCI (6 rats per group). The treatment group was observed blind by two independent observers, and the rats were scored according to the criteria for assessing spinal cord injury function proposed by Basso et al. The subtle movements and strength of the toes and feet of the rats were observed and recorded (Nikon, Japan). Then, the temperature of the body and hind limbs of the rat was measured by infrared thermal imaging (PORTIR, China). After painting the hind paws with black ink, the rats were allowed to run in a narrow dark track (10 cm x 50 cm) lined with paper, which was collected and used for footprint analysis.

### Histological Analysis

On week 5 after SCI, the spinal cord around the injury site (2 cm in length) was carefully separated and fixed in 4% paraformaldehyde (Beyotime, China), and then made into paraffin sections (5 µm in thickness). Paraffin sections were baked at 60 °C for 30 min, dewaxed with xylene and ethanol, and then rehydrated. The sections were used for hematoxylin‐eosin (HE) staining, immunohistochemical staining, and masson staining (Haoke Biotechnology, China).

### Hydrogel Swelling Profile

After the initial weight (*W*_0_) of the hydrogel was recorded, the hydrogel was soaked in PBS at 37 °C for 1 d, 2 d, and so on, measuring the weights (*W*1, *W*2, etc.) until swelling equilibrium was reached, defined as the point at which the difference between consecutive weightings was less than 0.01 g, and calculated the swelling rate (SR) using the following equation: SR = (*W*_n_‐*W*_0_)/*W*_0_*100%.

### Hydrogel Biodegradability

The in vivo degradation was probed by subcutaneously injecting 10 µL hydrogel samples within SD rats. The changes of hydrogel were observed at 0, 15, and 30 d, respectively. The in vitro degradation experiments of the hydrogel were conducted by soaking about 50 mg hydrogel in 1 mL of Artificial Cerebrospinal Fluid buffer (aCSF, Yuanye, China) at 37 °C. The hydrogel samples were weighed at 7, 14, 21, and 28 d respectively to analyze the degradation rate.

### Statistical Analysis

All experiments were performed at least three times. Data were expressed as the mean ± SD of all independent experiments. All of the statistically significant differences among various treatments were determined using Student's t‐test. Significance was set to *p < 0.05, **p < 0.01, ***p < 0.001, ****p < 0.0001 and ****p < 0.00001.

## Conflict of Interest

The authors declare no conflict of interest.

## Supporting information



Supporting Information

## Data Availability

The data that support the findings of this study are available in the supplementary material of this article.
