# Supplementary material for: Genetically Programmed Single‐Component Protein Hydrogel for Spinal Cord Injury Repair
Source: Adv Sci (Weinh). 2025 Jan 10;12(10):2405054. doi: 10.1002/advs.202405054 (PMC11904991; doi:10.1002/advs.202405054)
Supplement: Supplementary file 1 — Supporting Information [file ADVS-12-2405054-s001.pdf]

ADVANCED SCIENCE
Open Access

Supporting Information

for *Adv. Sci.*, DOI 10.1002/advs.202405054

Genetically Programmed Single-Component Protein Hydrogel for Spinal Cord Injury Repair

Yi Wei, Xiaolin Zhou, Zhenhua Li, Qing Liu, Han Ding, Yunlong Zhou, Ruo-feng Yin* and Lifei Zheng**

Supporting Information

Genetically Programmed Single-component Protein Hydrogel for Spinal Cord Injury Repair

Yi Wei [1,#], Xiaolin Zhou [1,#], Zhenhua Li [1], Qing Liu [1,], Han Ding [1], Yunlong Zhou [1], Ruo-feng Yin [2,*] and Lifei Zheng [1,*]*

Y. Wei, X. Zhou, Z. Li, Q. Liu, H. Ding, Y. Zhou, L. Zheng

Wenzhou Institute, University of Chinese Academy of Sciences, Wenzhou, Zhejiang, 325001, China.

E-mail: liuqing@ucas.ac.cn; zhenglf@ucas.ac.cn

R. Yin

China-Japan Union Hospital, Jilin University, Changchun, Jilin, 130031, China.

E-mail: yrf_wind@jlu.edu.cn

# These authors contributed equally to this work.

Materials

GdnHCl, NaCl, K_2HPO_4, KH_2PO_4, LB medium, IPTG, agar, and ampicillin were purchased from Solarbio Life Sciences Co. Ltd. (Beijing, China). SDS-PAGE reagent, coomassie brilliant blue dye and the reagents used in the WB process were purchased from Beyotime Co. Ltd. (Beijing, China). Cell culture plates and confocal dishes were were purchased from Nest Co. Ltd. (Wuxi, China). The deionized water was purified by using a BioSafer Millipore water purification system (Nanjing, China) .

Supplementary figures

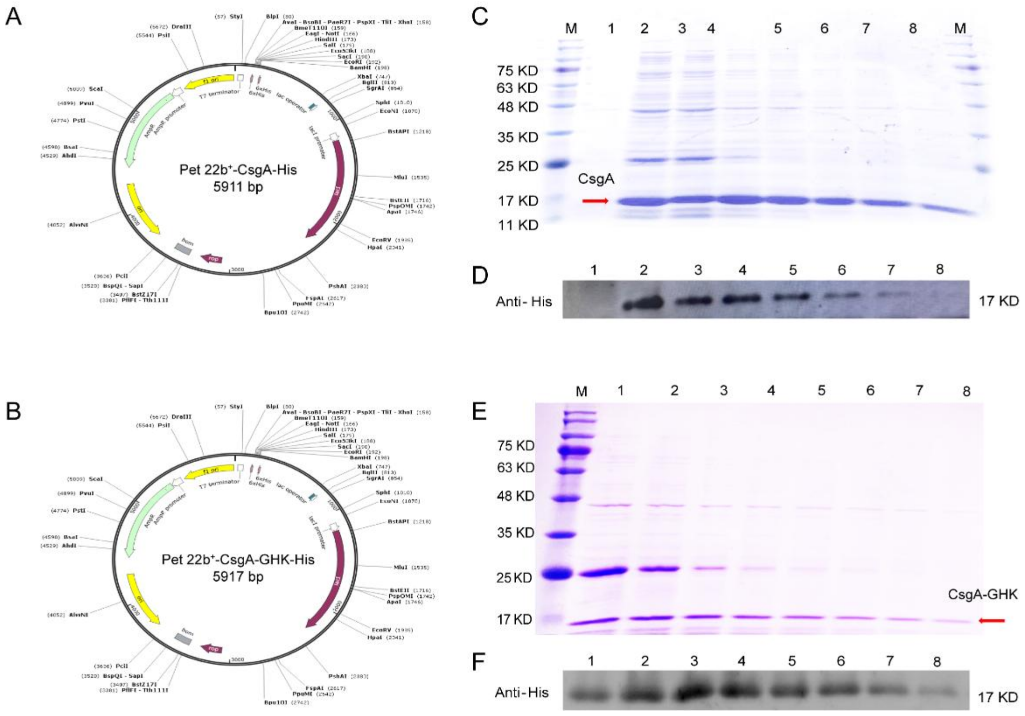

Figure S1. Plasmid profile and protein characterization. (A) Plasmid map of recombinant CsgA protein. (B) Plasmid map of recombinant CsgA-GHK protein. (C) SDS-PAGE analysis of recombinant CsgA protein purification. (D) WB result of anti-His for confirming the target protein. (E) SDS-PAGE analysis of recombinant CsgA-GHK protein purification. (F) WB result of anti-His for confirming the target protein.

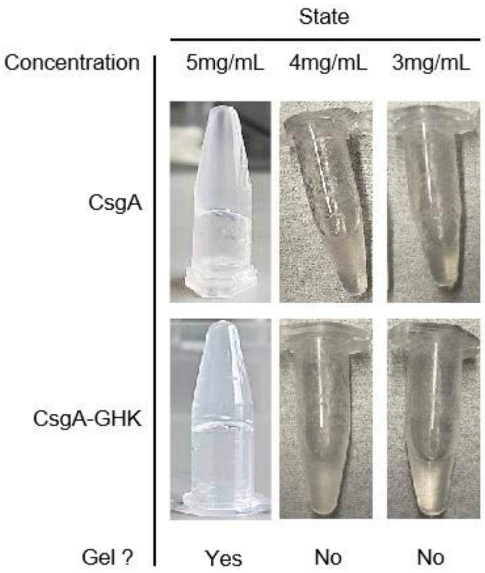

Figure S2. Photograph showing the gelation of proteins at different concentrations.

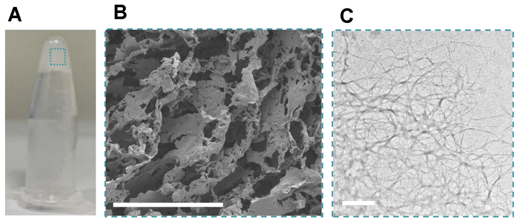

Figure S3. Characterization of CsgA gel. (A) Photograph of gelation of CsgA protein. (B) SEM image of CsgA hydrogel. Scale bars: 500 μm. (C) TEM image of CsgA nanofiber. Scale bars: 500 nm.

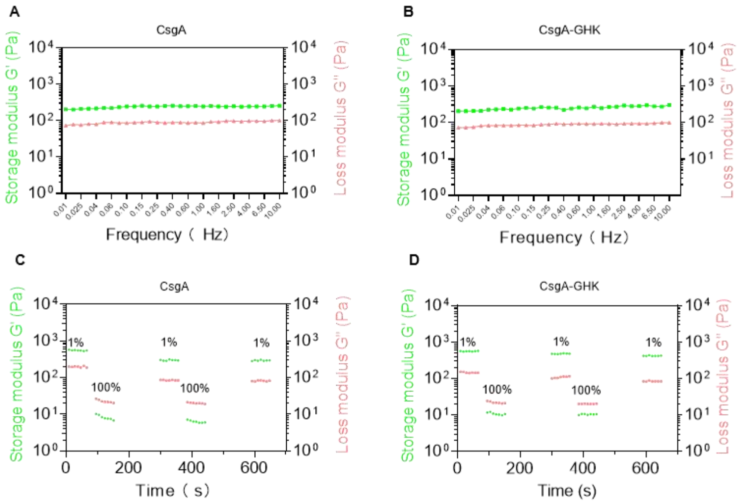

Figure S4. Rheological characterization of the protein hydrogels. (A, B) The changes in the modulus (G' and G") of the CsgA and CsgA-GHK hydrogels as a function of frequency at a fixed strain (γ) of 10% over time. (C, D) The changes in the modulus

(G' and G") of the CsgA and CsgA-GHK hydrogels at two fixed strains (γ) of 1% and 100% over time.

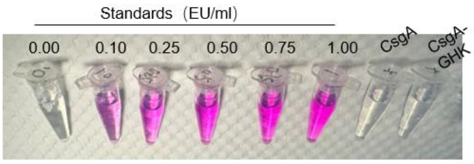

Figure S5. The endotoxin level of CsgA/CsgA-GHK hydrogel.

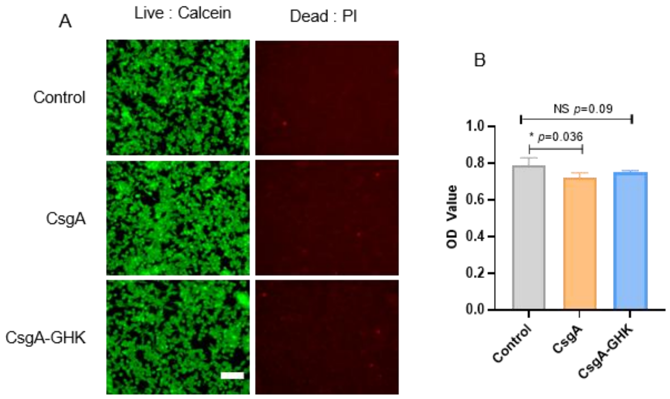

Figure S6. Cytotoxicity and cell viability detection of BV2 cell. (A) Live/Dead analysis of BV2 cells cultured on CsgA and CsgA-GHK gels. Green IF represents live cell marker Calcein while red IF represents dead cell marker PI. Scale bars: 100μm. (B) CCK8 analysis of BV2 cells cultured on CsgA and CsgA-GHK gels (n=3). Data are presented as mean ± s.d. ns, $p \geq 0.05$; *, $p < 0.05$.

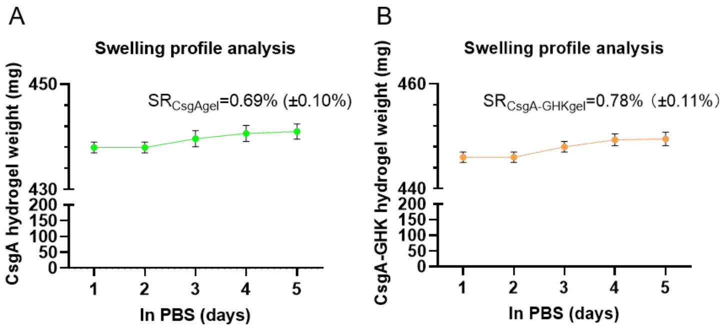

Figure S7. (A) The swelling profile of CsgA hydrogel (n=3). (B) The swelling profile of CsgA-GHK hydrogel (n=3).

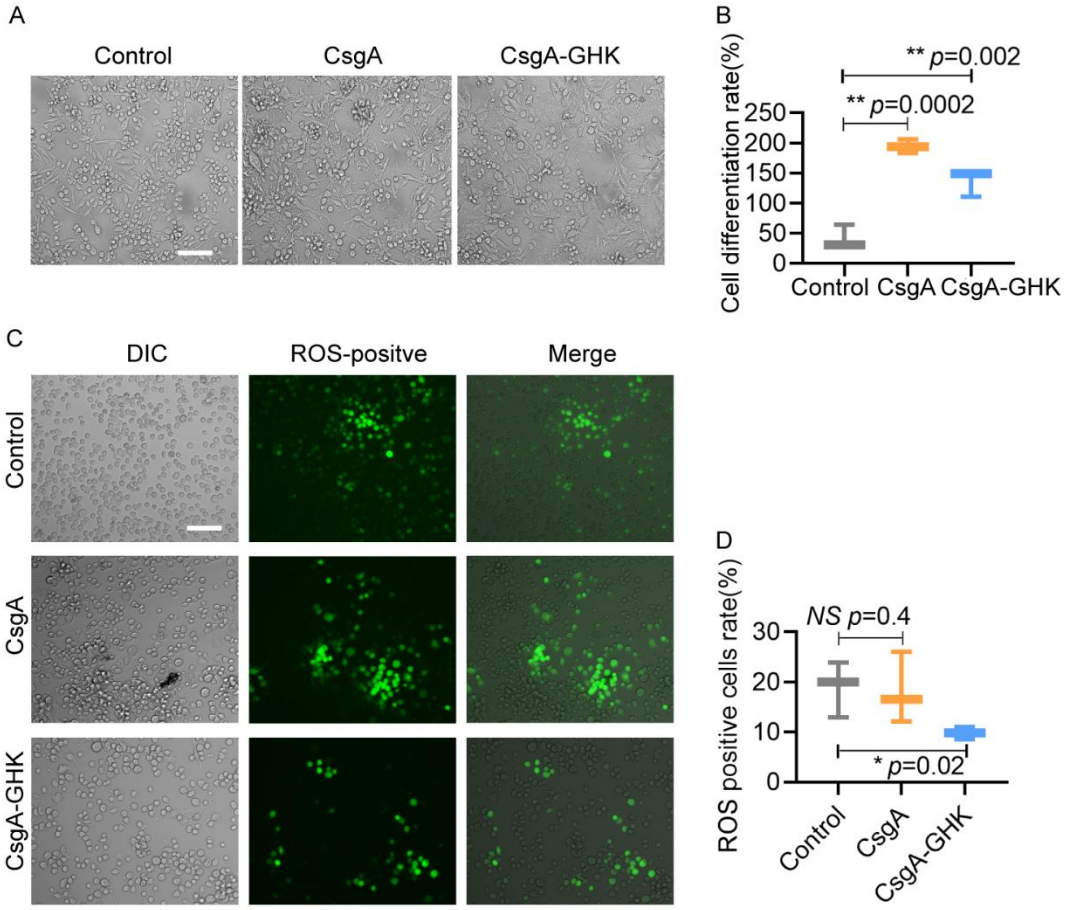

Figure S8. Determination of the differentiation and ROS production behavior of BV2 cells on CsgA and CsgA-GHK hydrogels. (A) Differentiation status of BV2 cells on CsgA and CsgA-GHK hydrogels. Scale bars: 100μm. (B) Statistics of BV2 cell differentiation (n = 3). (C) ROS positive BV2 cells cultured on each experimental group that was marked by green fluorescence. Scale bars: 100μm. (D) Quantification of ROS-positive cells in (C) (n = 3).

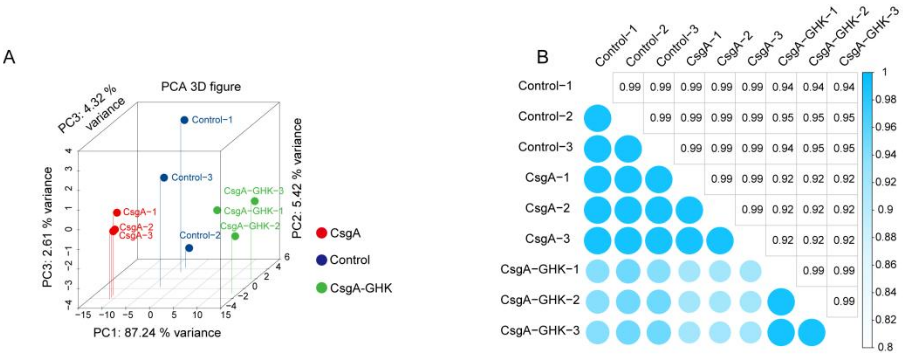

Figure S9. The sample repeatability analysis of RNA-seq data. (A) RNA-seq data

Principal Component Analysis (PCA). (B) Heat map of correlation coefficient between samples.

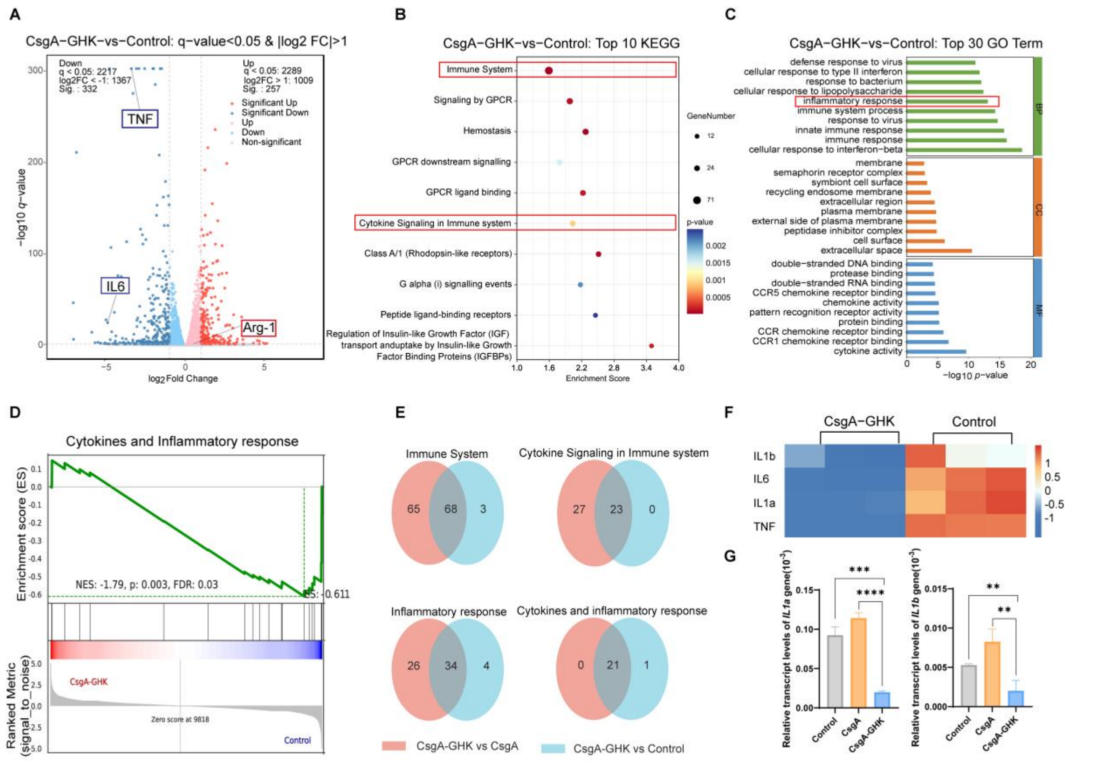

Figure S10. The RNA-seq of BV2 cells of CsgA-GHK vs Control group. (A) The volcano plots of identified up-regulated and down-regulated genes. (B) The KEGG pathway classification of significant differential genes (p < 0.05, fold change > 2). The red boxes mark the signal pathways of interest. (C) The GO pathway enrichment analysis of significant differential genes (p < 0.05, fold change > 2). The red box marks the signal pathway of interest. (D) The GSEA enrichment analysis of cytokines and inflammatory response pathway (p = 0.003). (E) The venn graphs of the four screened signal pathways of CsgA-GHK vs CsgA and CsgA-GHK vs Control groups for screening of common genes, respectively. (F) The heat map of *IL1b*, *IL6*, *IL1a*, and *TNF* genes expressions. (G) The gene expressions of *IL1a* and *IL1b* detected by RT-qPCR.

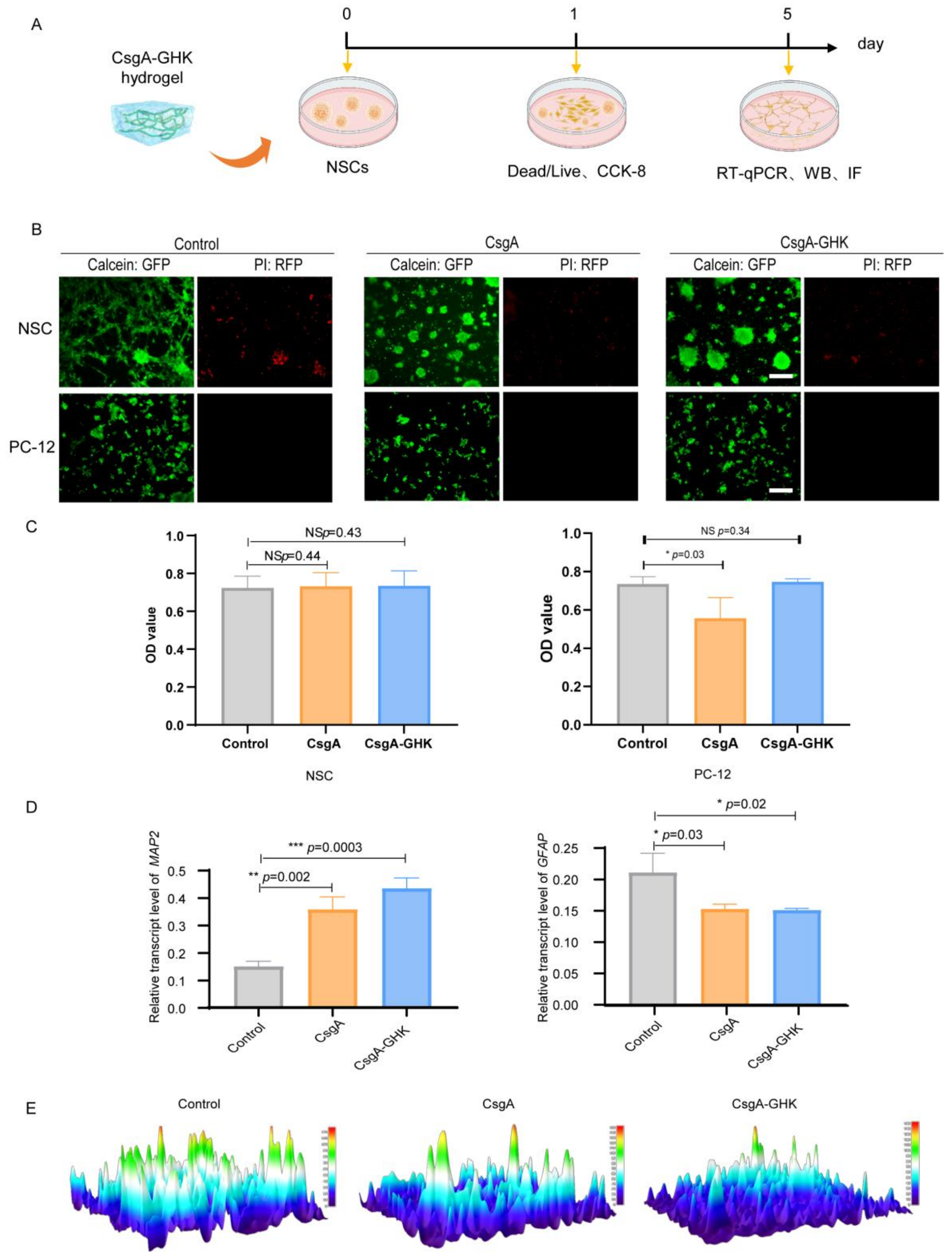

Figure S11. Biocompatibility analysis. (A) The experimental timeline schematics. (B) Live/Dead analysis of NSCs and PC-12 cells cultured on CsgA and CsgA-GHK gels. Green IF represents live cell marker Calcein while red IF represents dead cell marker PI. Scale bars: 200 nm. (C) CCK8 analyzes of NSCs and PC-12 cells cultured on CsgA and CsgA-GHK gels. (D) The RT-qPCR data showing CsgA and CsgA-GHK could induce GFAP expression and promote MAP2 expression (n = 3). (E) The 3D heat maps showed the expression of GFAP.

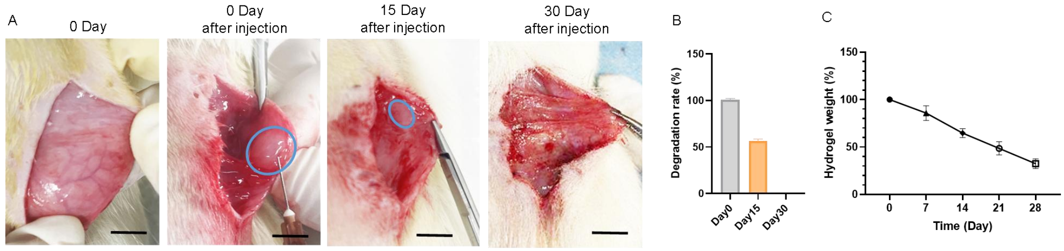

Figure S12. Degradability analysis of CsgA-GHK hydrogel. (A) Images showing the *in vivo* degradation of the CsgA-GHK hydrogel over time. Blue circles indicate the muscle areas where the hydrogel was injected. Scale bars: 1 cm. (B) Statistics on the degradation rate, with normalization treatment applied to the injected hydrogel area (n=3). (C) The degradation profile of the CsgA-GHK hydrogel in aCSF buffer over time (n=3).

Table S1. Protein sequences

Name	Sequence
CsgA-6×His	MKLLKVAAIAAIVFSGSALAGVVPQYGGGGNHGGGGNNSGPNSELNIYQYGGGNSALALQTDARNSDLTITQHGGGNGADVGQGSDDSSIDLTQRGFGNSATLDQWNGKNSEMTVKQFGGGNGAAVDQTASNSSVNVTQVGFGNNATAHQYGGGGSGGGGSGGGGSHHHHHH
CsgA-GHK-6×His	MKLLKVAAIAAIVFSGSALAGVVPQYGGGGNHGGGGNNSGPNSELNIYQYGGGNSALALQTDARNSDLTITQHGGGNGADVGQGSDDSSIDLTQRGFGNSATLDQWNGKNSEMTVKQFGGGNGAAVDQTASNSSVNVTQVGFGNNATAHQYGGGGSGGGGSGGGGSGHKHHHHHH

Table S2. The qRT-PCR primer sequences

Primer	Sequence (5'-3')
CsgA-F	CCCAAGCTTATGAAACTTTTAAAAGTAGCAGC
CsgA-R	CCGCTCGAGTTAATGATGATGATGATGATGGTACTGATGAGCGGTCGC
CsgA-GHK-F	CCCAAGCTTATGAAACTTTTAAAAGTAGCAGC
CsgA-GHK-R	CCGCTCGAGTTAATGATGATGATGATGATGTTTGTGACCGCTACCGCCACC
IL-6-F	GATTGTATGAACAGCGATGATGC
IL-6-R	AGAAACGGAACTCCAGAAGACC
TNF-α-F	GGGCTCTGAGGAGTAGACGATAAAG
TNF-α-R	GGGCAGGTCTACTTTGGAGTCATTG

iNOS-F	GTTCTCAGCCCAACAATACAAGA
iNOS-R	GTGGACGGGTCGATGTCAC
IL-10-F	CCAAGCCTTATCGGAAATGA
IL-10-R	TTTTCACAGGGGAGAAATCG
Arg-1-F	CTCCAAGCCAAAGTCCTTAGAG
Arg-1-R	GGAGCTGTCATTAGGGACATCA
CD206-F	CAAGGAAGGTTGGCATTTGT
CD206-R	CCTTTCAGTCCTTTGCAAGC
MAP2-F	ACAGAGAAACAGCAGAGGA
MAP2-R	GTTCACCTTTCAGGACTGC
GFAP-F	GAGTGGTATCGGTCCAAGTT
GFAP-R	CTCAAGGTCGCAGGTCAA
IL1a-F	AGATGCCTGAGATACCCAAAACC
IL1a-R	CCAAGCACACCCAGTAGTCT
Il1b-F	CCCAACTGGTACATCAGCACCTCTC
IL1b-R	CTATGTCCCGACCATTGCTG
GAPDH-F	TCCTGCACCACCAACTGCTTAG
GAPDH-R	AGTGGCAGTGATGGCATGGACT

104
